# A review of neuroendocrine immune system abnormalities in IBS based on the brain–gut axis and research progress of acupuncture intervention

**DOI:** 10.3389/fnins.2023.934341

**Published:** 2023-03-09

**Authors:** Zhangyin Sun, Xuejiao Wang, Shangsheng Feng, Chaoju Xie, Yu Xing, Liang Guo, Jingyu Zhao, Changchun Ji

**Affiliations:** ^1^College of Acupuncture and Moxibustion, Shaanxi University of Traditional Chinese Medicine, Xianyang, China; ^2^Department of Acupuncture and Moxibustion, Shaanxi Hospital of Traditional Chinese Medicine, Xi'an, China; ^3^MOE Key Laboratory of Biomedical Information Engineering, Xi'an Jiaotong University, Xi'an, China; ^4^Department of Acupuncture and Moxibustion, Xi'an Hospital of Traditional Chinese Medicine, Xi'an, China; ^5^Department of Acupuncture and Moxibustion, Shaanxi Provincial Institute of Traditional Chinese Medicine, Xi'an, China

**Keywords:** irritable bowel syndrome, brain-gut axis, neural signal regulation, endocrine system, immune system, acupuncture

## Abstract

Irritable bowel syndrome (IBS) is a common digestive disorder observed in clinics. Current studies suggest that the pathogenesis of the disease is closely related to abnormal brain–gut interactions, hypokinesia, visceral sensory hypersensitivity in the gastrointestinal tract, and alterations in the intestinal microenvironment. However, it is difficult for a single factor to explain the heterogeneity of symptoms. The Rome IV criteria emphasized the holistic biologic-psycho-social model of IBS, suggesting that symptoms of the disease are closely related to neurogastroenterology and various abnormalities in brain–gut interaction. This study comprehensively reviewed the relationship between the brain–gut axis and IBS, the structure of the brain–gut axis, and the relationship between the brain–gut axis and intestinal microenvironment, and discussed the relationship between the abnormal regulation of the nervous system, endocrine system, and immune system and the incidence of IBS on the basis of brain–gut axis. In terms of treatment, acupuncture therapy can regulate the neuroendocrine-immune system of the body and improve the intestinal microenvironment, and it has the advantages of safety, economy, and effectiveness. We study the pathogenesis of IBS from local to global and micro to macro, and review the use of acupuncture to treat the disease as a whole so as to provide new ideas for the treatment of the disease.

## 1. Introduction

Irritable bowel syndrome (IBS) is a complex functional gastrointestinal disorder characterized by abdominal pain, abdominal distension, and intestinal disorders that do not involve structural and biochemical abnormalities of the gastrointestinal tract (Ford et al., 2020). The Rome IV criteria classify patients with IBS into four subtypes based on their abnormal bowel movement traits: diarrheal IBS (IBS with diarrhea, IBS-D), constipated IBS (IBS with constipation, IBS-C), mixed IBS (IBS-M), and unclassified IBS (IBS-U) (Drossman, 2016). The global prevalence of IBS is approximately 11.2 % (Lovell and Ford, 2012). IBM seriously affects the quality of life of patients due to its high prevalence, recurrent symptoms, and susceptibility to complications, such as peptic ulcers, chronic liver disease, depression, and anxiety (Lai et al., 2021). In addition, it has led to a significant increase in the global healthcare burden (Black and Ford, 2020; Sperber et al., 2021).

The pathogenesis of IBS is multifactorial, which also has an important role in gene regulation, but the specific pathogenesis has not been fully elucidated. The disease is thought to be closely associated with abnormal brain–gut interactions, gastrointestinal hypokinesis, visceral sensory hypersensitivity, and intestinal microenvironmental disorders, indicating that a single factor cannot adequately explain the complexity of IBS symptoms. Therefore, treatment is mostly focused on improving gastrointestinal symptoms for different subtypes of IBS, currently involving analgesics, prokinetics, antidiarrheal agents, and psychotherapy (Ford et al., 2018; Arokiadoss and Weber, 2021). This can temporarily alleviate symptoms, but recurrence and varying degrees of side effects can occur (Aziz and Simrén, 2021), which are of great concern for clinical practitioners. Therefore, a fuller understanding of the pathogenesis of IBS is necessary to seek targeted therapies.

As IBS research progressed, it was defined by Rome IV as a disorder of gut–brain interactions (Schmulson and Drossman, 2017). For the understanding of the etiology and clinical features of IBS, Rome IV emphasizes a holistic biopsychosocial model, suggesting that the development of IBS symptoms is closely related to neurogastroenterology and multifaceted abnormalities in brain–gut interaction (Drossman and Hasler, 2016). The brain–gut axis maintains central and local homeostasis in the gut by integrating the neural, endocrine, and immune systems to form a bidirectional regulatory pathway and by connecting closely with the gut microbiota. Abnormalities in any of the brain–gut pathways may lead to the disruption of homeostatic balance and eventually induce IBS (Person and Keefer, 2021), in which neurological, endocrinological, and immunological factors intersect to produce symptoms that are the characteristic manifestation of the disease (Buckley et al., 2016). Therefore, understanding IBS from the perspective of abnormal regulation of the neuroendocrine-immune system in the brain–gut axis can facilitate the understanding of its pathogenesis.

## 2. Brain–gut axis

The brain–gut axis is a bidirectional information exchange system that integrates brain and intestinal functions, thereby effectively maintaining homeostasis in these organs (Carabotti et al., 2015). Different studies have confirmed that abnormal brain–gut interactions are closely related to IBS. Therefore, a deep understanding of the brain–gut axis would facilitate the elucidation of the pathogenesis of IBS. In the following sections, we highlight the basic architecture of the brain–gut axis and subsequently explore the relationship between the brain–gut axis and the intestinal microenvironment.

### 2.1. Basic architecture of the brain–intestine axis

Brain–gut interactions occur not only directly through neural pathways but also indirectly through humoral pathways (Bercik et al., 2012). First, the neural pathway of brain–gut interaction is a bidirectional interaction pathway linking the central nervous system (CNS) and the enteric nervous system (ENS). It is generally accepted that the regulation of gastrointestinal motility by the nervous system is achieved through three-level coordinated actions. Level 1 is the local regulation of the ENS, which consists of two plexuses: the interosseous plexus, which innervates the motility of the intestinal smooth muscle, and the submucosal plexus, which regulates intestinal mucosal sensation, secretion, and absorption. The sensory neurons, interneurons, and motor neurons of the ENS are interconnected to form separate, independent functions that integrate and process information similar to those of the brain and the spinal cord. Level 2 occurs in the anterior vertebral ganglion, which receives and regulates information from both the ENS and the CNS. Level 3 occurs in the CNS, which is composed of various centers at all levels of the brain and the spinal cord. They receive various incoming information during changes in the internal and external environment, integrate, and then transmit the regulatory information from the vegetative nervous and the neuroendocrine systems to the ENS, and act directly on gastrointestinal effector cells to regulate smooth muscle, glands, and blood vessels. This neuro-endocrine network linking the gastrointestinal tract to the CNS at different levels is the structural basis for the realization of brain–gut axis function (Bonaz and Bernstein, 2013).

Second, the humoral pathway mainly involves two bidirectional regulatory pathways and brain–gut peptides. Among them, the bidirectional regulatory pathways, namely, the hypothalamic-pituitary-adrenal axis (HPA) and the hypothalamic-autonomic nervous system axis (HANS), are the main pathways involved in physiological and psychological stress responses (Stasi et al., 2012).

In addition, brain and intestinal peptides are hormones that regulate the movement of the gallbladder and bile ducts, such as gastrokinetic hormone, cholecystokinin, and glucagon. These peptides are highly distributed in the gastrointestinal and nervous systems, and hence, they are called brain and intestinal peptides. Brain and intestinal peptides of the gastrointestinal and nervous systems regulate the complex functions of the gastrointestinal tract, such as movement, sensation, secretion, and absorption through endocrine, neurosecretory, and paracrine secretory peptides (Wu I. X. Y. et al., 2019). Different types of intestinal peptides, such as 5-hydroxytryptamine (5-HT), substance P (SP), calcitonin gene-related peptide, vasoactive intestinal peptide, somatostatin, neuropeptide Y, and cholecystokinin, can act on the central nervous and the enteric nervous systems, such that both systems can jointly regulate the functions of the gastrointestinal tract. In addition, different brain and intestinal peptides have different mechanisms of action, and most brain and intestinal peptides do not act on IBS in isolation. There are complex interconnections between various brain and intestinal peptides, which are jointly involved in the regulation of the physiological activities of the gastrointestinal tract.

### 2.2. Relationship between the brain–gut axis and the intestinal microenvironment

**Figure 1:**
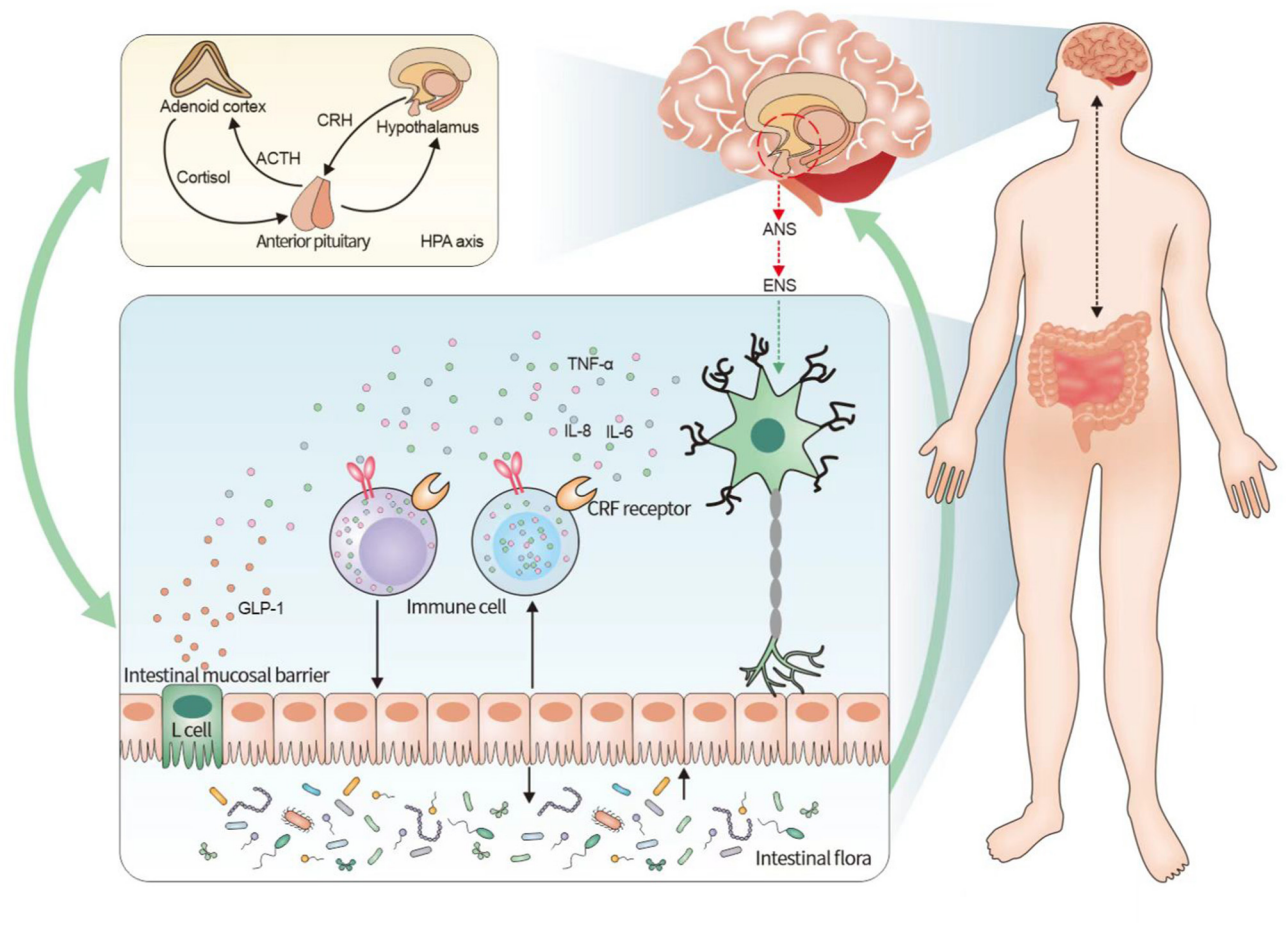
Relationship between irritable bowel syndrome and the neuroendocrine-immune system based on the brain–gut axis. The figure illustrates that the pathogenesis of irritable bowel syndrome can be understood in terms of the neuroendocrine-immune network system of the brain–gut axis. The brain–gut axis connects the ENS and the CNS and activates the endocrine system mainly by initiating the HPA axis in the CNS, while the endocrine system has a bidirectional regulation with the intestinal immune system, and the intestinal mucosal barrier, immune cells, and intestinal flora are involved in the intestinal immune system, among which the intestinal flora forms another bidirectional regulation with the brain–gut axis. It indicates that the neuroendocrine-immune system of the brain–gut axis influences the pathogenesis of IBS, and the three systems form a multidimensional and bidirectional regulation among themselves. CRF, adrenocorticotropin-releasing factor; HPA, hypothalamus-pituitary-adrenal; IBS, irritable bowel syndrome.

In the brain–gut interaction, neurological dysfunction can lead to alterations in the intestinal microenvironment (Li et al., 2020). This is composed of the microbiota, local immune system, and epithelium of the single-cell layer, which can effectively regulate homeostasis in the body and consequently maintain normal physiological functions of the gastrointestinal tract (Fay et al., 2017). Therefore, the relationship between the brain–gut axis and the intestinal microenvironment was explored from three perspectives: the intestinal microbiota, intestinal immune system, and intestinal mucosal permeability (the relationship between the brain–gut axis and the intestinal flora is shown in Figure 1).

In recent years, studies on the pathogenesis of IBS have focused on the brain–gut axis, intestinal microecological relationships (Hillestad et al., 2022), and intestinal microbiota disorders (Aziz et al., 2017). The brain–gut axis integrates the neural, endocrine, and immune systems, forming a bidirectional regulatory pathway that maintains the homeostasis of brain and gut functions. The gut microbiota is an important player in the brain–gut bidirectional information exchange system. On the one hand, its interaction with the ENS and neurological and endocrine signaling pathways profoundly affects the brain–gut communication function (Martin et al., 2018); and on the other hand, it can influence the brain–gut axis function through direct and body circulation pathways. This indicates that the gut microbiota and the brain–gut axis are closely linked and interact with each other, thereby forming a complex brain–gut microbiota network to maintain normal gut function.

The brain–gut axis can influence the host gut flora, and similarly, the gut microbiota can effectively regulate some of the functions of the brain–gut axis (Khlevner et al., 2018). First, the brain–gut axis can influence the intestinal microbiota. On the one hand, the response signals from the CNS are transmitted to the smooth muscle layer of the intestine through the relevant fibers, thereby influencing gastrointestinal motility and secretion and regulating the intestinal flora environment (Mayer et al., 2014). On the other hand, stress and emergency stimulation of the host CNS will have an impact on the intestinal flora. It has been reported that neuroinflammation induced by traumatic brain injury through the brain–gut pathway results in impaired intestinal motility and increased intestinal mucosal permeability (Hanscom et al., 2021). Second, the intestinal microbiota can maintain the functional homeostasis of the host gut and participate in the regulation of neurological development as well as mood, appetite, cognition, and behavior in the host (Mu et al., 2016). The decrease in the number and diversity of gut flora is closely associated with neurological dysfunctions in the host, such as abnormalities in mood and behavior. Through the blood circulatory pathway, gut microbiota and its metabolites, such as short-chain fatty acids, neurotransmitters, and their precursors can influence the expression levels of neurotransmitters, precursors, and their receptors in the CNS, thus regulating brain function, cognition, and behavior (Cryan et al., 2020). Moreover, improving the disturbance of the intestinal microbiota and reconfiguring intestinal homeostasis may facilitate the recovery of the neurological function of an organism (O'Hagan et al., 2017). Bidirectional communication takes place between the brain and the gut, and the involvement of the gut microbiota has recently been discovered. An abundance of symbiotic microorganisms inhabit the lumen of the gastrointestinal tract and signal the immune and endocrine systems of the host through the secretion of metabolites such as short-chain fatty acids (SCFAs), amino acids, and polyamines. The aim of our study was to investigate specific types of microbiota-derived metabolites, specifically bile acids, short-chain fatty acids, vitamins, amino acids, serotonin, and hypoxanthines, which are all implicated in the pathogenesis of IBS.

Dysregulation of the microbial-gut-brain axis can exacerbate various CNS disorders through the expression of abnormal metabolites, such as SCFAs. Microbiota-derived metabolites play a central role in the communication between microbes and their hosts, with SCFAs probably being the most studied.

Short-chain fatty acids (SCFAs), mainly derived from the fermentation of dietary fiber, play a key role in host gut metabolism and immune function. Stress increases fecal acetate and total SCFA levels as well as colonic *FFAR2* and *FFAR3* expressions. Moreover, SCFA supplementation ameliorated acute stress-induced elevated body temperature and corticosterone levels in chronically stressed mice. Similar changes were found in colonic MR gene expression, although stress-induced increases in hippocampal MR gene expression were not affected. In addition, no differences in GR gene expression were found in any of the tissues investigated, and these differences may have disappeared over time. Sustained decreases in *CRFR1, CRFR2*, and *MR* expressions were observed in the group that received SCFA injections alone, and SCFA also had antidepressant and anxiolytic effects (Van De Wouw et al., 2018). Overall, these results suggest that SCFAs can down-regulate stress signaling and HPA axis responses, a critical pathway for microbe-gut-brain axis communication (Wiley et al., 2016). Studies using germ-free (GF) mice lacking all microbes provide compelling and consistent evidence that the microbiota is essential for brain development. GF mice exhibit altered expression of neurotransmitters and their receptors and neurotrophic factors in the brain, exhibit region-specific gene expression, and have an impaired blood–brain barrier. Even hippocampal neurogenesis and neuroplasticity in mice were affected by the absence of the gut microbiota. The maturation of oligodendrocytes is inhibited by specific gut microbial metabolites that alter myelin formation patterns in the limbic system. Therefore, a healthy, stable, and diverse intestinal microbiota is essential for normal brain–gut interactive communication.

In addition, the CNS directly affects the intestinal microbiota through stress-mediated virulence gene expression and indirectly through ANS-mediated intestinal motility, intestinal immune regulation, and secretion (Osadchiy et al., 2019). The top-down effect of the brain on the gut microbiota was also confirmed in a recent clinical randomized controlled trial (Jacobs et al., 2021).

The CNS damage increases intestinal mucosal permeability. For example, a large release of norepinephrine from cecum sympathetic nerves, altered cecal mucin production, and cupped cell numbers were observed in rats with brain injury, and this affected the intestinal microbiota and permeability (Houlden et al., 2016). We reviewed the literature and found that ANS dysfunction is seen after brain injury, and this exaggerates sympathetic activation (Purkayastha et al., 2019). In addition, under stressful stimuli, large amounts of neurotransmitters and receptors, such as norepinephrine and catecholamines, are released in the peripheral circulation (Zhang et al., 2020), and these regulate intestinal mucosal permeability.

Furthermore, the close relationship between HPA axis activation, a key humoral pathway in the brain–gut axis, and intestinal permeability has been demonstrated in clinical trials (Arciniega-maptinez et al., 2022). First, adrenocorticotropin-releasing factor (CRF) released by the HPA axis can increase intestinal permeability (Lyte et al., 2011). Second, the gut microbiome involves the blood–brain barrier (BBB) in its interactions with the peripheral and neuroimmune systems, and on the BBB, the gut microbiome can interact with the CNS and regulate body function. Bacteria can release the microbiome and its factors directly into the body's circulation and bloodstream. Once in the blood, the microbiome and its factors can alter peripheral immune cells to facilitate interaction with the BBB and eventually with other elements of the neurovascular unit. Bacteria and their factors or cytokines and other immunologically active substances released from peripheral sites under the influence of the microbiome can cross the BBB, alter the integrity of the BBB, change the rate of BBB transport, or induce the release of neuroimmune substances from barrier cells (Logsdon et al., 2018). In addition, metabolites produced by the microbiome, such as short-chain fatty acids, can cross the BBB to affect brain function. Through these and other mechanisms, microbiome–BBB interactions can influence the disease process, as reported for multiple sclerosis (Dopkins et al., 2018). Preclinical studies provide evidence that the BBB is altered when animals are subjected to experimental intestinal infections or when animals lack a normal gut microbiome.

In addition, the ENS has an important role in gastrointestinal peristalsis, digestion, secretion, and regulation of intestinal mucosal permeability (Furness, 2012). The ENS plays a key role in regulating pH in the gastrointestinal lumen and consequently intestinal inflammation, which is strongly associated with increased mucosal permeability (Tanaka et al., 2016).

With the exploration of the gut–brain axis, it was found that alpha synucleinopathy can be transmitted in both directions in the brain and the intestine. The pathology of PD [Parkinson's disease (PD)] is characterized by alpha synucleinopathy, which proves that the brain–gut axis also plays an important role in the pathogenesis of PD. In addition, the presence of a large microbiota in the gut, which is involved in the formation and transmission of alpha synucleinopathy, suggests that dysbiosis of the gut microbiota is closely related to the progression of PD.

## 3. Abnormal neural signal regulation

The cerebro-intestinal axis is a neuroendocrine network that connects the gastrointestinal system with the CNS, and it involves three main nervous systems: the CNS, ANS, and ENS. The gastrointestinal tract is the only organ in the body that is innervated by the CNS, ANS, and ENS, and its functional activities are neurologically regulated by these three systems. The impaired functions of any of the systems can cause gastrointestinal tract dysfunction and mediate the development of IBS.

### 3.1. ANS dysfunction

The ANS is part of the peripheral efferent nervous system that regulates the activity of the visceral and vascular smooth muscle, the cardiac muscle, and the glands. The sympathetic and parasympathetic nervous systems, under the control of the cerebral cortex and the hypothalamus, both antagonize and coordinate the physiological activities of the organs. The structure of the autonomic nervous system (ANS) can be divided into central and peripheral parts. The ANS is mainly distributed in the visceral, cardiovascular, and glandular bodies, whose central part is also located in the brain and the spinal cord. The peripheral part includes visceral motor (efferent) and visceral sensory (afferent) fibers, which constitute the visceral motor and visceral sensory nerves, respectively.

The ANS plays an important role in the brain–gut axis as a bridge between the central and the enteric nervous systems; hence, it is able to regulate the movement and sensation of the intestine. There are changes in autonomic function in patients with IBS that may be associated with autonomic dysfunction (Salvioli et al., 2015). The ANS comprises two nervous systems, sympathetic and parasympathetic, whose structures and functions are integrated into the brain–gut axis and work together to maintain functional homeostasis in the intestine. On the one hand, the sympathetic nervous system inhibits gastrointestinal motility and secretion, while the parasympathetic nervous system plays a stimulatory and pro-secretory role in smooth muscle. Sympathetic–parasympathetic imbalance causes altered bowel habits in patients with IBS. On the other hand, the modulation of visceral sensitivity by both is closely related to abdominal pain in IBS (De Winter et al., 2016). The parasympathetic afferent pathway begins in the vagus nerve and ends in the nucleus tractus solitarius, which transmits pain signals to the corticolimbic layer. The sympathetic afferent pathway mediates pain signals primarily through the spinal cord, first to the thalamus and then to the sensory cortex and pain stroma. The signal also travels to specific areas of the brain, such as the hippocampus, the amygdala, the prefrontal cortex, and finally to the hypothalamus (Fukudo, 2013). In addition, important tissues and organs in the CNS can interact with the HPA axis and the ANS. These central regions, which regulate intestinal function, are involved in both emotions (e.g., mood, anxiety, and pain) and cognitive behaviors (e.g., memory and decision-making), such that the dysfunction of the ANS directly or indirectly controls the development and progression of IBS disease.

### 3.2. CNS dysfunction

The CNS is composed of the brain and the spinal cord (the brain and the spinal cord are the central parts of various reflex arcs) and is the most dominant part of the human nervous system. It receives afferent information from all parts of the body, which is integrated and processed into coordinated motor efferents or stored in the CNS to become the neural basis for learning and memory.

Brain dysfunction has an important relationship with the development of IBS. Some key sites in the CNS associated with emotion-pain regulation are structurally altered in patients with IBS (Aziz and Simrén, 2021). For example, Sheehan et al. (2011) found a correlation between the activity of the cingulate gyrus and the pain-related cerebral cortex in patients with IBS and the clinical symptoms of IBS. Increased neuronal activity has been reported in the medial prefrontal cortex, the insula, and the anterior cingulate gyrus, which are brain regions associated with visceral pain in patients with IBS (Grinsvall et al., 2021), and a larger area of excitation was observed in brain regions associated with nociceptive processing (e.g., the prefrontal cortex, the anterior cingulate gyrus, the thalamus, etc.,) (Seminowicz et al., 2010). Similarly, in a cohort study, the IBS group exhibited extensive structural changes in the brain compared with that in the controls, and these changes in the brain included a reduction or increase in gray matter volume in multiple regions of the sensorimotor, central executive, and default mode networks, and these changes were strongly associated with chronic visceral pain (Öhlmann et al., 2021). Another study found that spontaneous activity in the amygdala and the anterior insula was significantly increased in patients with IBS, while spontaneous activity in sensorimotor areas tended to reduce (Hong et al., 2013). Moreover, cognitive-behavioral treatment reduced activity in the anterior cingulate gyrus, the amygdala, and the hypothalamus, suggesting that spontaneous activity of the brain may be associated with some symptoms of IBS. In addition, patients with IBS have smaller volumes in the bilateral superior frontal gyrus, the bilateral insular gyrus, the bilateral amygdala, the left cingulate gyrus, the left rectus gyrus, the left shell nucleus, and the brainstem, and larger volumes in the left postcentral gyrus (Labus et al., 2014), which are mostly part of the limbic system and are crucial for the body to perceive visceral injurious stimuli, pain production, emotional processing, and emotion regulation. Thus, CNS abnormalities can lead to visceral pain and emotional abnormalities in patients with IBS.

### 3.3. ENS dysfunction

The ENS consists of the ganglia contained in the gastrointestinal tract, the biliopancreatic system, and the network between them, which can connect the gastrointestinal tract with the CNS and ANS. On the one hand, it transmits sensory information to the brain through neurons, nerve fibers, and neurotransmitters connected to the central nerve, such that the activities of the intestine are regulated by the center. On the other hand, it has considerable autonomic functions to locally regulate the motility, secretion, and blood flow, as well as water and electrolyte transport of the intestine. The ENS consists of the ganglia and the biliopancreatic system contained in the gastrointestinal tract as well as the network between the ganglia and biliopancreatic system that connects the gastrointestinal tract to the CNS and ANS.

On the one hand, it transmits sensory information to the brain through neurons, nerve fibers, and neurotransmitters connected to the central nerve, such that the activities of the intestine are regulated by the center. On the other hand, it has considerable autonomic functions to locally regulate the motility, secretion, blood flow, as well as water and electrolyte transport of the intestine.

When the intestine is stimulated by mechanical dilatation, stress, inflammation, and allergens, chromophores release 5-HT as a paracrine signal acting on intestinal mast cells, spinal afferent cells, and neurons in the ENS. Intestinal mast cells release multiple mediators of paracrine signaling, including histamine and serine proteases, chymotrypsin and trypsin, and serotonin. Some of these mediators diffuse to receptors at the afferent injury-sensitive and mechanosensitive end of the spectrum and are known to cause abdominal pain and distention that may lead to IBS (Wood, 2020). In addition, 5-HT acts on spinal afferent neurons to mediate central responses to intestinal stimuli, as well as on 5-HT receptors on enteric neurons to influence intestinal sensory and motor functions. In IBS-D model rats, the total number of the ganglia and neurons in the submucosal plexus of the small intestine increased, the proportion of excitatory neurons (cholinergic neurons and vasoactive intestinal peptide neurons) increased, and the proportion of inhibitory neurons (nitrogenergic neurons) in the intermuscular plexus decreased (Li et al., 2016). Such morphological changes in the ENS are consistent with accelerated intestinal transmission and increased secretion in IBS-D.

The enteric nervous system (ENS) contains many neurotransmitters or brain intestinal peptides and can regulate intestinal function through a variety of neurotransmitters. When the internal environment of the gastrointestinal tract is altered, the intrinsic primary sensory neurons can be modified to produce strong sensory signals and transmitted upward to cause discomfort in the body. Therefore, the activation of primary neurons in the enteric plexus is one of the mechanisms associated with visceral hypersensitivity in IBS. Studies on enteric neurotransmitters and their complex signaling pathways as well as the interaction between enteric neurons and peripheral glial cells and Cajal mesenchymal cells have led to a better understanding of the functional regulation of the ENS in the pathogenesis of IBS (Zhang et al., 2019; Matheis et al., 2020). Therefore, ENS abnormalities are associated with the development of IBS.

## 4. Endocrine system abnormalities

The brain–gut axis has an important role in the development of IBS, and patients with IBS have hyperfunction of the HPA axis and impaired function of the endocrine system, which leads to abnormalities in an organism. Although there are many factors that affect the endocrine system, those with important roles include adrenocorticotropin-releasing factor (CRF), corticosteroids, and glucagon-like peptide 1 (GLP-1).

### 4.1. CRF

Adrenocorticotropin-releasing factor (CRF) is an important hormone associated with the body's response to stress. It is a major mediator of stress responses in the brain–gut axis, while the HPA axis is key to maintaining homeostasis and is responsible for various responses in the endocrine system. CRF plays a key role in the activation of the HPA axis under basal and stressful conditions (Chen et al., 2012). It may also explain the higher levels of adrenocorticotropic hormone and cortisol in patients with IBS, compared with that in healthy subjects (Distrutti et al., 2016). CRF exerts its biological effects by activating CRF1 and CRF2 receptors (CRFR1 and CRFR2), stimulating colonic motility, and inducing visceral hypersensitivity responses (Nozu and Okumura, 2015). CRFR1 is commonly found in the regions of the brain associated with pressure and nociceptive circuits, including the paraventricular nucleus, the ventricles, and the amygdala (Reyes et al., 2008). Toll-like receptor 4 (TLR4) or IL-1 receptor antagonists block abnormal visceral pain and increase intestinal permeability caused by CRF (Nozu et al., 2018). CRF receptor (CRF-R) signaling has critical roles in stress-related alterations in gastrointestinal function, stimulation of the colonic enteric nervous system, secretory motor function, intestinal permeability, and visceral hypersensitivity (Tache et al., 2018). In addition, Labus et al. found that peripheral administration of CRF1 receptor antagonists in patients with IBS reduced abdominal pain and anxiety (Labus et al., 2014). In addition, CRF receptors were found in interosseous nerves, sensory nerves, sympathetic nerves, intestinal chromophores, and immune cells in the guts of animals and humans. This suggests that the central and peripheral CRF system regulates the body's response to stress and modulates the syndromes that occur in IBS (Chen et al., 2012; Nozu and Okumura, 2015). In the immune context, CRF and CRF receptors are present in immune cells in the intestine. Stress, through the local CRF system, activates intestinal immune cells, leading to intestinal pro-inflammatory cytokine release, increased permeability, mucin secretion, and visceral allergy (Chatoo et al., 2018).

### 4.2. Corticosteroids

Corticosteroids are steroids produced by the adrenal cortex, and they mostly include hormones, such as glucocorticoids, mineralocorticoids, and sex hormones. They are readily absorbed by the gastrointestinal tract and are highly bound to proteins and play an important role in the treatment of intestinal disorders (Kapugi and Cunningham, 2019). Salt corticosteroids (MR) and glucocorticoids (GR) are steroid hormones that act in the stress system model through salt corticosteroid and glucocorticoid receptors (MRs and GRs, respectively), mediating the roles of adrenal hormones, such as cortisol, in the initiation and termination of the stress response, respectively. Corticosterone, which is the stress hormone in the amygdala, induces visceral hypersensitivity through the actions of GR and MR (Myers and Greenwood-Van Meerveld, 2012). It has been demonstrated that higher cortisol levels in patients with IBS positively correlated with psychological stress levels (Videlock et al., 2016). In addition, glucocorticoids, including endogenous cortisol, inhibit the production and activity of many inflammatory cells, as well as redistribute them to other parts of the body, resulting in fewer circulating immune cells (Ericson-Neilsen and Kaye, 2014). Chronic stress induces alterations in the expression of GR and CRH, leading to increased visceral pain and colonic permeability, while exposure to environmental enrichment suppresses stress-induced changes within the brain–gut axis to prevent visceral and somatic hypersensitivity and colonic hyperpermeability (Orock et al., 2021). All these indicate that the central signaling of corticosteroids is a potential target for the treatment of intestinal dysfunction in IBS.

### 4.3. GLP-1

Ingestion of certain foods is a predisposing factor for certain IBS symptoms (Simren et al., 2001). It has been shown that reducing the consumption of fermentable oligosaccharides, disaccharides, monosaccharides, and polyols (FODMAPs) may alleviate the symptoms of bloating, abdominal pain, and osmotic diarrhea in IBS (Barrett et al., 2010; Zahedi et al., 2018). Intestinal secretion of glucagon is an important physiological response that occurs in the intestine after eating. The secretion of glucagon-like peptide 1 (GLP-1) by L cells, expression of the surface receptor TGR5 by intestinal L cells, and induction of GLP-1 release from enteroendocrine cells by the TGR5 agonist 6α-ethyl-23(S)-methylcholic acid (EMCA, INT-777) inhibits glucagon secretion and gastric emptying and suppresses food uptake (Thomas et al., 2009). One study demonstrated that the glucagon-like peptide-1 receptor agonist ROSE-010 reduced pain during IBS episodes (Touny et al., 2022). However, GLP-1 acts on the enteric nervous system by reducing excitatory cholinergic neurotransmitters and regulating nitric oxide release through presynaptic GLP-1Rs (Amato et al., 2010). In addition, GLP-1 can initiate colonic enteric neurons and the vagus nerve (Amato et al., 2010); the vagus nerve is involved in the pathophysiological processes of IBS; hence, GLP-1 may influence the central regulation of visceral pain sensitivity. Interestingly, GLP-1 also modulates cytokines secreted by the GI tract and alters the central CRF pathway that regulates stress-induced alterations in colonic transit (Nakade et al., 2007).

## 5. Abnormal immune system

Current studies have confirmed the existence of an immune-activated state and the involvement of brain–gut interaction in the development of IBS. The immune system is an essential link in the neuroendocrine-immune network system, and IBS acts through immune cells, immune factors, and intestinal mucosal immune aspects and thus affects the whole system.

### 5.1. Immunocytes

Mast cells (MC), an intrinsic component of the neuroimmune axis, are present at elevated levels in IBS mucosal samples (Buhner and Schemann, 2012; Lee and Lee, 2016; Krammer et al., 2019). Although other immune cells may be involved in the pathophysiological process of IBS (Uranga et al., 2020), mast cells are important cellular mediators of local nerve fiber sensitization (Casado-Bedmar and Keita, 2020). The chronic inflammation of the intestinal mucosa in patients with IBS increases intestinal epithelial permeability. This leads to increased exposure of intestinal mucosa to intestinal contents, causes mast cell activation and degranulation to release large amounts of inflammatory mediators (Nogueira et al., 2017), stimulates visceral sensory neurons, causes the release of neuropeptides, and participates in pathophysiological processes, such as pain perception and cognition, through HANS upregulation mode (Skaper et al., 2014; Mackey et al., 2016). Similarly, mast cells can be activated by neuropeptides, such as SP, leading to the release of protein hydrolases, as a stress response to pain. Therefore, IBS is considered a brain–gut axis disease (Casado-Bedmar and Keita, 2020; Labanski et al., 2020). IBS biopsies showed an increased number of mast cells in the lamina propria, compared with that observed in the healthy controls (Krammer et al., 2019), and increased concentrations of products secreted by mast cells, such as histamine, proteases, cytokines, and prostaglandins, in patients with IBS activate intestinal neurons (Balestra et al., 2012). In addition, MC activation is associated with CRF, and it leads to increased visceral hypersensitivity and intestinal permeability by acting on mast cells and causing degranulation and release of trypsin-like enzymes, tumor necrosis factor-α, and histamine (Zhang et al., 2016; Tache et al., 2018). This effect may be due to the involvement of neuroendocrine-immune interaction mechanisms in the pathogenesis of IBS (Jarret et al., 2020).

In addition, the feedback response of the immune system induced by the HPA downregulation pattern can cause low-grade inflammation after which the colon is more susceptible to the effects of stress on intestinal nerve function (Wouters et al., 2016). The activated immune system in patients with IBS, as evidenced by increased expression of colonic mucosal cytokines and increased release of pro-inflammatory cytokines from monocytes isolated from peripheral blood, especially in patients with IBS-D, is specific to the gastrointestinal tract (Vicario et al., 2015; Van De Wouw et al., 2018). Increased humoral immunoreactivity in the jejunal mucosa of patients with IBS-D was identified by microarray analysis, which correlated with the activation of B lymphocytes and the production of immunoglobulins.

### 5.2. Immune factors

Evidence of immune activation in IBS includes elevated levels of pro-inflammatory cytokines, such as interleukin-6 (IL-6), IL-8, and tumor necrosis factor-α (Brzozowski et al., 2016; Seyedmirzaee et al., 2016; Ivashkin et al., 2021). On the one hand, the cytokines have neuromodulatory effects and can stimulate the excitation of intestinal mucosal pro-secretory neurons, which can also lead to changes in intestinal functions (e.g., contraction, absorption, and secretion). In addition, IL-6 releases the sympathetic brake by suppressing the presynaptic inhibition of norepinephrine release. On the other hand, visceral pain can be evoked after cytokine excitation of enteric neurons (Buckley et al., 2016), making cytokines an important cause of visceral pain-mediated IBS (Atmaramani et al., 2020).

It has been reported that the levels of anti-inflammatory cytokines (e.g., IL-10 and transforming growth factor beta) decreased in IBS colon and rectal biopsies (Coëffier et al., 2010; Ivashkin et al., 2021). Probiotics, as living microorganisms, can modulate the immune system, leading to the upregulation of anti-inflammatory cytokines and growth factors, and can interact with the brain–gut axis by regulating endocrine and neurological functions (Atmaramani et al., 2020). Thus, immune factors play an important role in the pathogenesis of IBS as intermediate transmitters or different “links” in the regulation of the neuroendocrine-immune network.

### 5.3. Intestinal mucosal immunity

The intestinal mucosal immune system is composed of intestinal epithelial cells, intestinal interepithelial lymphocytes, lymphocytes of the lamina propria, intestinal submucosal-collecting lymph nodes, and various monocytes, which directly participate in intestinal immune regulation. Some studies have confirmed the presence of abnormal mucosal immunity in patients with IBS (Aerssens et al., 2008), and stress factors are thought to be strongly associated with the development of IBS (Elsenbruch and Enck, 2016). Stress regulates the intestinal immune system by triggering the brain–gut axis to produce neurotransmitters and hormones. Secretory immunoglobulin A (SIgA) is the most important immunoglobulin on the mucosal surface of the intestine, and stress can cause the upregulation or downregulation of SIgA levels, thus affecting intestinal immunity (Campos-Rodríguez et al., 2013). SIgA production is regulated by stress (Brawner et al., 2020) and pIgR expression (Jarillo-Luna et al., 2015), and the immunoglobulin-pIgR complex (Diga-pIgR and PIGA-pIgR) is transported cellularly through the intestinal epithelium. Upon reaching the tip of these cells, pIgR is cleaved, releasing sIgA, and a pIgR-derived polypeptide into the intestinal lumen (Brandtzaeg, 2013). Therefore, SIgA and pIgR protect the intestinal epithelium from pathogens and regulate the intestinal inflammatory response to maintain homeostasis *in vivo*. In addition, visceral nerve fibers are distributed in the submucosa (Meissner's plexus) and mucosal plexus, which contain nerve endings that can contact antigen-presenting cells to control intestinal immune responses (De Jonge, 2013; Jacobson et al., 2021). These indicate that the IBS mucosal immune system also plays a significant role in the CNS.

## 6. Discussion

Irritable bowel syndrome (IBS) is a common functional gastrointestinal disorder that has a significant impact on the quality of life of patients due to its high prevalence, severity, and recurrence; hence, effective interventions are urgently needed. The efficacy of current interventions does not meet the clinical requirements of patients because the pathophysiology of the disease is not fully understood in terms of etiology and heterogeneity of symptoms. Therefore, it is necessary to have a comprehensive understanding of the disease toward the development of targeted interventions for improved clinical treatment.

Under the social–psychological–biological model advocated by Rome IV, this study highlights the pathogenesis of IBS from the perspective of abnormal regulation of the neuroendocrine-immune system of the brain–gut axis. In addition, it finds that a complex system consisting of several systems, such as the nervous, immune, and endocrine systems, participates in the regulation of the brain–gut axis. IBS may occur if there is a problem in a certain link, which may also trigger the dysfunction of other systems, resulting in the diversity and severity of IBS symptoms.

Traditional Chinese medicine is a medical theory system gradually formed and developed in long-term medical practice. In the long-term clinical use of TCM against digestive system diseases, we have discovered a close relationship between the brain and the intestine. Interestingly, this relationship is validated by the brain–gut axis theory from current microscopic studies, which suggest that there is a theoretical basis for the efficacy of TCM acupuncture therapy against IBS. Modern studies have shown that TCM acupuncture therapy can effectively regulate the functional levels of the brain–gut axis in rats with IBS (Sun et al., 2015), suggesting that TCM acupuncture therapy is a potential complementary alternative therapy for IBS.

In recent years, acupuncture therapy has attracted the attention of medical researchers in China and abroad because of its safety, efficiency, and green features. In particular, its efficacy in regulating gastrointestinal dysfunction and extraintestinal conditions has been favored by clinical medical practitioners worldwide. A large number of clinical studies have confirmed that acupuncture therapy contributes to the management of IBS, such as systematic evaluation studies demonstrating the effectiveness of acupuncture therapy (Wu Y. et al., 2019) and randomized controlled clinical trials investigating its feasibility and efficacy (Pei et al., 2020; Qi et al., 2021; Shen et al., 2022).

In addition, different studies have confirmed that acupuncture can modulate IBS from the intestinal and dorsal root ganglion perspectives (Jin et al., 2021), brain functional interconnections and effects (Ma et al., 2020), and at the level of the brain–gut axis (Zhu et al., 2017), and expert treatment consensus opinions have been formed (Zhu et al., 2017). In conclusion, acupuncture therapy can effectively treat IBS and modulate gastrointestinal symptoms and concomitant mood disorders in patients.

Irritable bowel syndrome (IBS) is a complex functional gastrointestinal disease, and it is difficult to dissect the complexity of the disease from the perspectives of abnormal brain–gut interactions, hypokinetic and visceral sensory hypersensitivity, and altered intestinal microenvironment alone. Rather, clinical workers can understand IBS from a holistic bio-psycho-social model, which has the following five guiding implications for patients and researchers: (1) the combination of anti-anxiety and depression drugs can be used to treat irritable bowel syndrome in clinical work, providing a new way of thinking for the clinical treatment of the disease; (2) the treatment can be done from the perspective of treating the bowel from the brain; (3) acupuncture can be used to treat irritable bowel syndrome from the aspect of anxiety and depression; (4) keeping patients away from stressors to avoid long-term stimulation can be a treatment method; and (5) patients can be provided with stress training, psychological training, and positive meditation to prevent later relapse or frequent relapse.

## Author contributions

CJ and JZ: study concept and design. ZS, XW, and CJ: search for information. ZS and XW: draft of the manuscript. All authors contributed to the article and approved the submitted version.
